# Supporting routine cognitive reactivity assessment during the perinatal period: psychometric testing of the Chinese version of the Leiden Index of Depression Sensitivity

**DOI:** 10.1186/s12884-022-05233-6

**Published:** 2022-12-06

**Authors:** Yanqing Fu, Yu-an Lin, Jiansheng Zheng, Huilan Hong, Songqing Huang, Jiang Li, Feifei Huang

**Affiliations:** 1 Obstetrics and Gynecology, Fujian Medical University Union Hospital Department of Surgical Nursing, Fujian Fuzhou, China; 2 School of Nursing, Fujian Medical University, 350108 Fuzhou, China; 3 School of Basic Medicine, Putian University, Putian, Fujian China; 4 Obstetrics and Gynecology, The Second Affiliated Hospital of Fujian Medical University, Quanzhou, Fujian China; 5 Obstetrics and Gynecology, Affiliated Hospital of Putian University, Putian, Fujian China; 6 Obstetrics and Gynecology, Fujian Provincial Maternity and Child Health Hospital, Fuzhou, Fujian China

**Keywords:** Cognitive reactivity, Perinatal care, Depression, Reliability and validity, Item response theory, Longitudinal study

## Abstract

**Background:**

It is critical to find optimal forms to identify perinatal depression (PND) and its vulnerable factors and make them more applicable to depression screening. This study aims to evaluate the reliability and validity of the Chinese version of the Leiden Index of Depression Sensitivity (LEIDS-RR-CV) among perinatal women in China and determine the cut-off values for screening for high-risk depression.

**Methods:**

Women in their third trimester of pregnancy and six weeks postpartum completed the LEIDS-RR-CV and a diagnostic reference standard online. We assessed the LEIDS-RR-CV using classical test theory (CTT) and item response theory (IRT). We also assessed the test performance for cut-off scores using receiver operator characteristic analysis to further screen for high-risk depression at each time point.

**Results:**

In total, 396 (third trimester) and 321 (six weeks postpartum) women participated. Cronbach’s alpha, two-week test–retest reliability, and marginal reliability for the scale were all greater than 0.8. It showed a five-factor model; the cut-off values were 58 (third trimester) and 60 (six weeks postpartum). The areas under the curve were acceptable (≥ 0.7), and the LEIDS-RR-CV was positively correlated with the total Edinburgh Postnatal Depression Scale (EPDS) score (*r* = 0.52 and 0.56, *p* = 0.00), indicating its predictive validity. An IRT analysis further confirmed its discriminative validity.

**Conclusions:**

The LEIDS-RR-CV was found to be reliable, valid, and can be used to quantify cognitive reactivity among perinatal Chinese women and for screening for high-risk depression during this period.

**Supplementary Information:**

The online version contains supplementary material available at 10.1186/s12884-022-05233-6.

## Background

The perinatal period is a vulnerable time for developing mental health disorders, including perinatal depression (PND) [1]. The fifth edition of the Diagnostic and Statistical Manual of Mental Disorders defines PND as the occurrence of a major depressive episode during pregnancy (i.e., antenatal depression: onset during pregnancy) or within four weeks after childbirth (i.e., postpartum depression [PPD]), and use the specifier ‘‘with peripartum onset’’ to define the depressive disorder [2].

During the last decade, the prevalence of PND has shown a significant increasing trend. In 2020, a systematic review in mainland China, which examined the prevalence of PND and its determinants, reported a 16.3%, 19.7%, and 14.8% pooled prevalence of PND, antenatal depression, and PPD, respectively [3]. PND entails various adverse health outcomes for both mothers and babies [1, 4, 5]. These include increased risk of preeclampsia, pregnancy, labor complications, infanticide or suicide for mothers and preterm births, low birth weight, and poor cognitive and emotional development for infants and newborns [1, 4, 5].

The Edinburgh Postnatal Depression Scale (EPDS) is the most widely used screening tool for women who experienced PND worldwide [6]. However, PND remains under-detected and under-treated worldwide, especially across developing countries [7, 8]. There are limited data on the diagnostic and treatment rates for PND in China. However, following the implementation of the three-child policy in China and the release of the “explore the work plan of characteristic services for the prevention and treatment of depression” blueprint, stakeholders have begun paying more attention to PND [9]. Therefore, consistent with previous research [10] and based on primary prevention, it is critical to find better ways to ascertain women at high risk of PND and its risk factors, especially during the risk period (prenatal period to one-year postpartum).

According to Beck’s cognitive model, one’s vulnerability to depression is marked by schemas or dysfunctional attitudes [11]. Teasdale (1988) explains that negative cognitions remain latent in some individuals but maybe (re) activated by life events, stress, or even negative moods [12], a concept known as cognitive reactivity (CR) [13]. Studies show that CR is a significant cognitive vulnerability factor for the onset and persistence of depressive symptoms, showing moderate to high predictive power [14, 15].

Some studies have used the Dysfunctional Attitudes Scale (DAS) to explore the relationship between dysfunctional attitudes and PPD and found that perinatal women with high dysfunctional attitudes are more vulnerable to PPD [16, 17] and that such attitudes are indirectly related to depression and anxiety symptoms among infants [18]. However, previous studies show that DAS has two versions with different forms but the same content (DAS-A and DAS-B). Therefore, both versions of the DAS need to be tested repeatedly before and after mood induction, which may be time-consuming, consequently affecting the comparability of the results and further inhibiting the differentiation of non-depressed and depressed patients [12, 13, 19]. Therefore, this may hinder the generalization of the findings.

To compensate for these limitations, the Leiden Index of Depression Sensitivity (LEIDS)—a self-reported inventory of CR that does not entail mood induction, was developed [13]. This scale was later revised to LEIDS-R [20] and LEIDS-RR [21], which have been translated into several languages and validated in many countries [15, 22], including China [23]. The Chinese version of the LEIDS-RR (LEIDS-RR-CV) is a reliable and valid instrument for evaluating CR among Chinese patients diagnosed with clinical depression in remission and the healthy Chinese population [14, 23].

To the best of our knowledge, no studies have conducted a psychometric analysis of the LEIDS-RR and applied it to perinatal women, especially in China. Therefore, this study aims to examine the psychometric performance of the LEIDS-RR-CV and explore its applicability to identify women at high risk of PND. Specifically, we use classical test theory (CTT) and item response theory (IRT) to conduct the psychometric analyses.

## Methods

### Participants and procedures

This prospective study was conducted between September 2020 and March 2021. Using convenience sampling, we recruited pregnant women who met the discharge standards of the obstetrical clinic and obstetrical inpatient department of four tertiary (level 3) hospitals in Fuzhou, Putian, and Quanzhou City, Fujian Province

The inclusion criteria for the women in the third trimester included (1) ages ≥ 20 years, (2) singleton, late pregnancy (i.e., gestational age ≥ 28 weeks) and with fetal survival diagnosed through a type-B ultrasonic test, (3) the possibility of being followed-up for up to six weeks postpartum, (4) voluntary participation in the study and signing the informed consent form, and (5) absence of depression (a score ≤ 12 on the 10-item EPDS) [24]. The exclusion criteria for women in the third trimester included (1) having severe mental disorders, (2) undergoing mental health treatment, (3) having cognitive impairment, and (4) having a serious physical illness. The exclusion criteria for the woman at 6 weeks postpartum included (1) malformation of fetus or infant, (2) death of the fetus or infant, and (3) significant negative stress events for the mother in the previous three months.

We determined the sample size based on a subject-to-item ratio of 5–10:1, assuming a non-response rate of 20%, and attained a final sample size of at least 320 women. Eligible women were asked to complete the study measures at both time points. They participated in both surveys through the Wenjuanxing online platform (a popular online survey platform in China, https://www.wjx.cn/m/91639653.aspx. / and https://www.wjx.cn/m/91655868.aspx.). At each time, women were able to opt out of the survey.

## Measures

The participants completed the following measures in the survey questionnaire at both assessment points.

### Chinese version of the Leiden Index of Depression Sensitivity (LEIDS-RR-CV)

We used the 26-item self-reported LEIDS-RR-CV, comprising five subscales: hopelessness/suicidality, acceptance/coping, aggression, control/perfectionism, and avoidant coping, to evaluate CR. Items were rated on a five-point Likert scale ranging from 1–5 (“not at all” to “very strongly”). After reverse scoring the acceptance/coping subscale and summing up the scores for each item, higher total scores indicated greater CR. The LEIDS-RR-CV has been validated and shown good psychometric properties; Cronbach’s alpha was 0.92 [23].

### Edinburgh Postnatal Depression Scale (EPDS)

We used the 10-item self-reported Edinburgh Postnatal Depression Scale (EPDS), which is the most widely used screening tool for common depression symptoms among pre- and postpartum women. Items were rated on a four-point Likert scale ranging from 0–3, with higher total scores indicating more depression symptoms (range: 0–30) [24]. The EPDS has been validated in several countries and shown good psychometric properties in both pre-and postpartum contexts [25]. When assessing the potential for PND, a cut-off score of 13 or more is recommended [24]. In the PND sample, Cronbach’s alpha was 0.87 and 0.90.

### Sociodemographic and clinical characteristics

We collected the sociodemographic and clinical information of the participants from the administrative database of the participating hospital; data included telephone number, maternal age, education level, only child or not, residential location, city, pregnancy intention, monthly household income (yuan, RMB), gravidity, parity, gestational week, and expected due date.

### Ethics

The study was approved by the appropriate ethical committee; all participants provided written informed consent.

### Statistical analysis

Analyses were conducted using IBM SPSS Statistics, version 26.0, AMOS 25.0, and Stata 14.0. If α = 0.05, the difference was considered statistically significant (*p* < 0.05). We conducted the psychometric analysis of LEIDS-RR-CV based on CTT and IRT for both assessment time points.

### CTT-based psychometric analysis

Regarding reliability, we used Cronbach’s alpha of the total scale to assess internal consistency. Test–retest reliability was evaluated using the intraclass correlation coefficient (ICC). Twenty-four more eligible women were included at the time points of the third trimester and six weeks postpartum when we conducted the assessments, with an interval of two weeks, respectively. We calculated the ICC between the test scores at both time points.

We further conducted confirmatory factor analysis (CFA) to analyze the structural validity of the tool using the maximum likelihood method. We evaluated the model’s goodness-of-fit using absolute and relative indices: normed χ2 (χ2/df; 1.0–3.0) and root mean square error of approximation (RMSEA; < 0.08); goodness-of-fit index (GFI), adjusted goodness-of-fit index (AGFI), comparative fit index (CFI), and Tucker–Lewis Index (TLI), all of which were > 0.9 [26].

We conducted a Spearman correlation analysis between the total score of the EPDS and LEIDS-RR-CV to analyze criterion-related validity. For known-group validity, 50 women in the third trimester and 42 women at six weeks postpartum who exhibited depressive symptoms, that is, EPDS score > 13, were selected by propensity score matching and completed the measure of the LEIDS-RR-CV. We further used independent t-tests to calculate the mean scores between participants that exhibited depressive symptoms and those that did not.

We determined predictive validity based on the EPDS criteria by calculating the receiver operating characteristic (ROC), the area under the curve (AUC), sensitivity, specificity, positive predictive value, negative predictive value, and percentage. These outcomes also determined the optimal cut-off points for LEIDS-RR-CV among childbearing and postpartum women.

### IRT-based psychometric analysis

We used Samejima’s graded response model to conduct the IRT analysis [27]. After meeting the assumption of unidimensionality [28], we calculated the discrimination parameter (*a*_*i*_), difficulty parameters (*β*_*i*_), item information value, item characteristic curves, and test information functions. Lastly, we determined reliability based on marginal reliability, the amount of information provided by individual items, and the entire scale.

## Results

### Participants’ characteristics

Four hundred and fourteen women participated in this study. Of these, 396 (20–42 years old) and 321 women (20–42 years old) completed the questionnaire during the third trimester and at six-weeks postpartum, respectively. Overall, the ratio of the women who met the EPDS criteria for depression at the two-time points accounted for 13.1% and 13.4%, respectively. Table 1 summarizes additional sociodemographic and clinical characteristics of the participants.

**Table 1:** The socio-demographic and clinical characteristics of participants

Variables	The third trimester women (*n* = 396) n(%)	The 6-week postpartum women (*n* = 321) n(%)
Educational level		
Junior high school or below	53(13.4)	43(13.4)
Middle school or Technical school	63(15.9)	47(14.6)
College or above	280(70.7)	231(72.0)
Only child or not		
Yes	72(18.2)	59(18.4)
No	324(81.8)	262(81.6)
Residential location		
Urban	296(74.7)	246(76.6)
Rural	100(25.3)	75(23.4)
City		
Fuzhou	174(43.9)	147(45.8)
Putian	71(17.9)	52(16.2)
Quanzhou	132(33.3)	115(35.8)
Other^a^	19(4.8)	7(2.2)
Monthly household income (yuan, RMB)		
< 3000	21(5.3)	14(4.4)
3000 ~ 4999	114(28.8)	89(27.7)
5000 ~ 8000	158(39.9)	136(42.4)
> 8000	103(26.0)	82(25.5)
Gravidity		
1	173(43.7)	145(45.2)
2	153(38.6)	132(41.1)
≥ 3	70(17.7)	44(13.7)
Parity		
0	201(50.8)	168(52.3)
1	157(39.6)	135(42.1)
≥ 2	38(9.6)	18(5.6)
Pregnancy intention		
In plan	262(66.2)	217(67.6)
Out of plan	134(33.8)	104(32.4)

Other^a^ include Xiamen, Ningde, Sanming, Longyan, Zhangzhou, Nanping and other cities

### Reliability

Data from women who responded at both time points had a total Cronbach’s alpha of 0.928 and 0.982, marginal reliability of 0.943 and 0.981, and ICC of 0.934 and 0.862, respectively, with the result showing that the test–retest reliability of the total scale was good.

### Structural validity

**Fig. 1:**
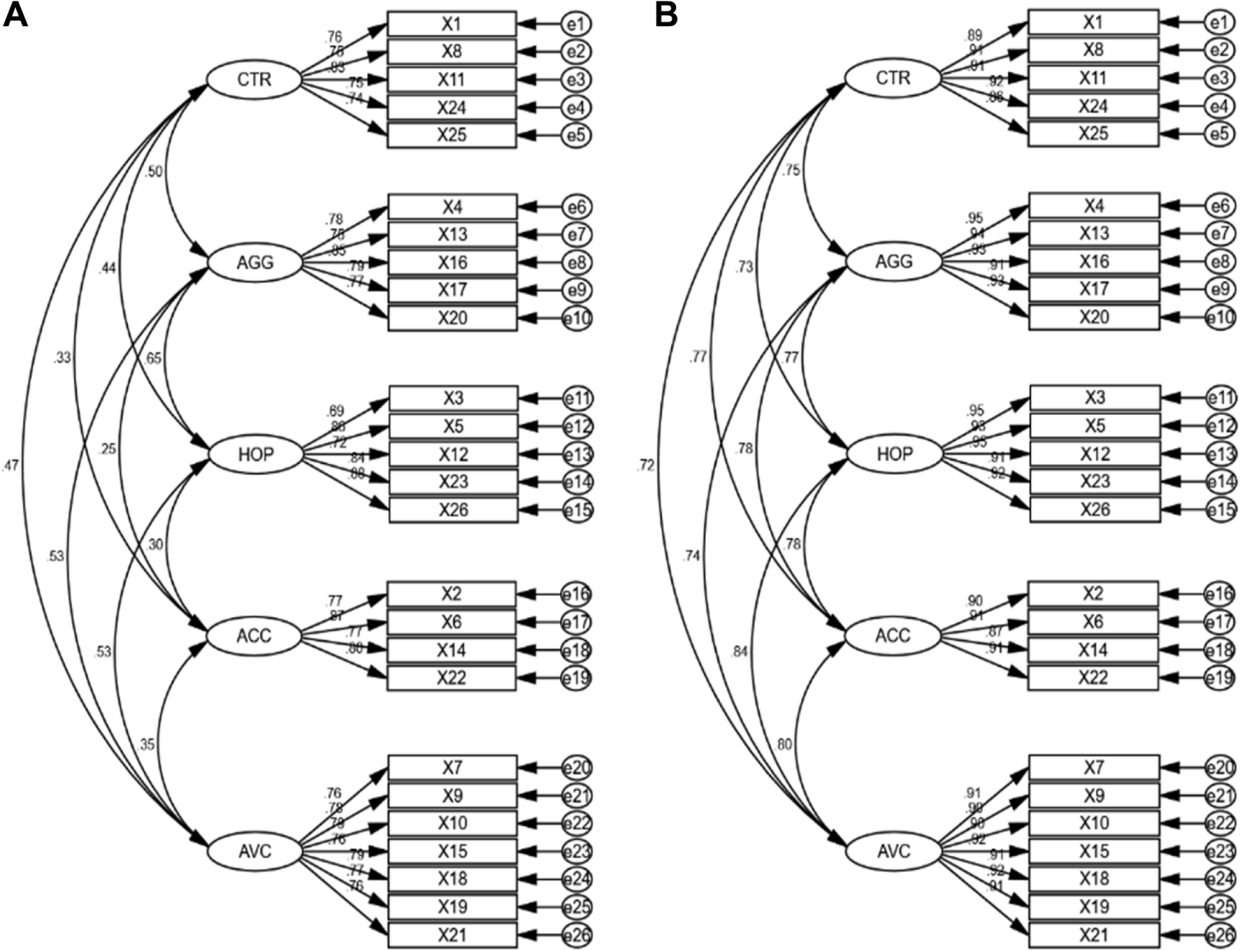
**A** The factor structure of LEIDS-RR-CV in women of late pregnancy. **B**. The factor structure of LEIDS-RR-CV in women of 6 weeks postpartum. Note. HOP: Hopelessness/suicidality. ACC: Acceptance/coping. AGG: Aggression. CTR: Perfectionism/control. AVC: Avoidant coping

Through CFA, we observed that a five-factor structure was a good fit to the data at both time points (χ2/df = 2.511 and 2.586, GFI = 0.861 and 0.840, AGFI = 0.830 and 0.803, RMSEA = 0.066 and 0.070, CFI = 0.925 and 0.963, and TLI = 0.915 and 0.957, respectively). The factors included (a) hopelessness/suicidality, (b) acceptance/coping, (c) aggression, (d) control/perfectionism, and (e) avoidant coping (Fig. 1).

### Criterion-related validity

The total score for the LEIDS-RR-CV showed a positive correlation with the total score for the EPDS (*r* = 0.52 and 0.56 in the third trimester and at six-weeks postpartum, respectively; *p* = 0.00).

### Known-group validity

Women with depression showed higher total scores for the LEIDS-RR-CV (third trimester: 69.08 ± 11.68; six-weeks postpartum: 74.88 ± 15.74) than women with no depression (53.68 ± 11.73 and 61.45 ± 14.84, respectively). The two groups differed significantly (t = 6.58 and 4.02, respectively; *p* < 0.001).

### Predictive validity

**Fig. 2:**
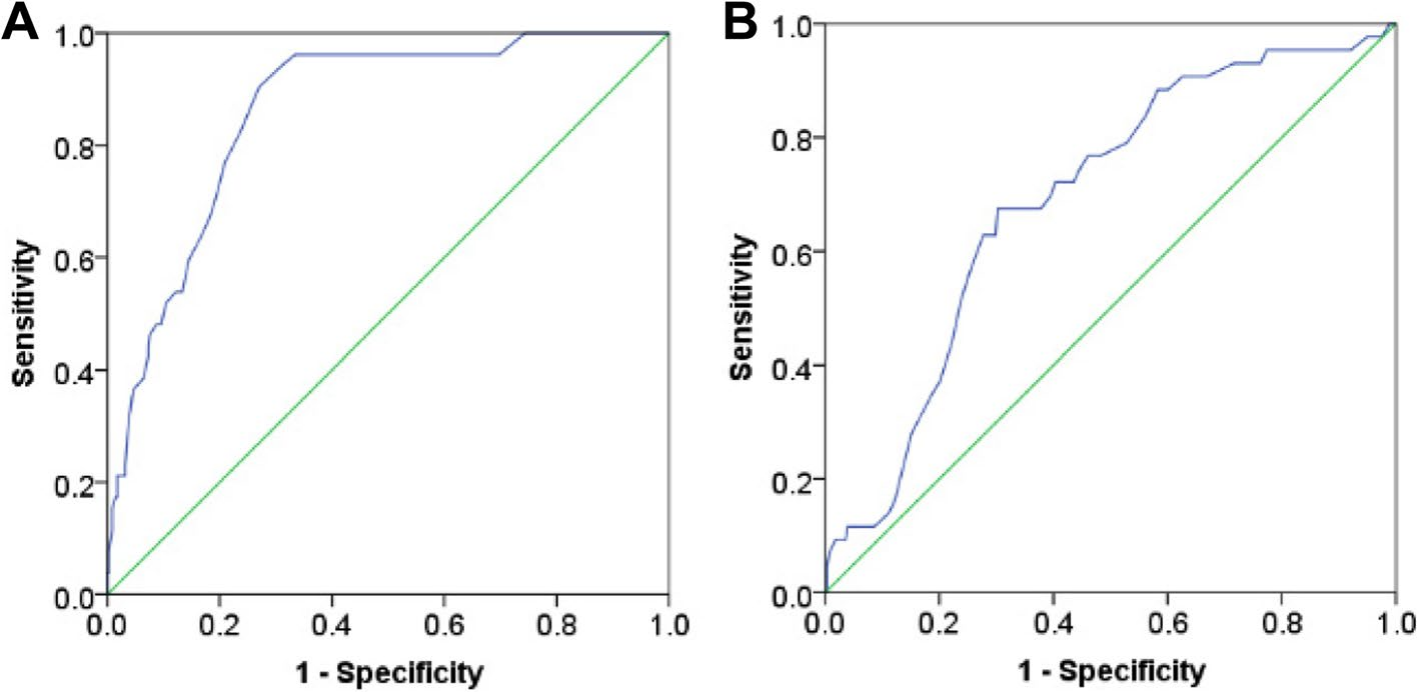
**A** The receiver operator characteristic curve of the LEIDS-RR-CV in women of late pregnancy. **B** The receiver operator characteristic curve of the LEIDS-RR-CV in women of 6 weeks postpartum. Note. LEIDS-RR-CV: the Chinese version of the modified Leiden index of depression sensitivity

The AUC of the ROC for the LEIDS-RR-CV of the data from participants at both time points was 0.861 (95% confidence interval [CI]: 0.815–0.908) and 0.691 (95% CI: 0.612–0.770) (Fig. 2), indicating good predictive power for depression.

In the third trimester, the optimal cut-off value for the LEIDS-RR-CV to screen for groups at high risk of depression was 58 points, and the sensitivity and specificity were 0.904 and 0.730, respectively. At six-weeks postpartum, the optimal cut-off value was 60 points, and the sensitivity and specificity were 0.767 and 0.540, respectively.

### The IRT analysis of LEIDS-RR-CV

We observed support for the unidimensionality assumption based on the percentage of variance accounting for the first factor (i.e., 37.59%: third trimester; 69.19%: six weeks postpartum), as well as the eigenvalues of the first factor divided by the second, which were 3.59 and 13.84. Table 2 shows the parametric estimates (*a, β*_*1*_*, β*_*2*_*, β*_*3*_*, β*_*4*_) for the 26 items from the IRT analysis.

**Table 2:** IRT parameter estimates for the LEIDS-RR-CV for perinatal women

Item	The third trimester women							The 6-week postpartum women						
	Slopeα(SE)	Difficulty				Maximum value of TIF	Mean value of TIF^a^	Slopeα(SE)	Difficulty				Maximum value of TIF	Mean value of TIF^a^
		β_1_ (SE)	β_2_ (SE)	β_3_ (SE)	β_4_ (SE)				β_1_ (SE)	β_2_ (SE)	β_3_ (SE)	β_4_ (SE)		
I1	1.12 (0.14)	-1.01 (0.16)	1.43 (0.19)	3.40 (0.45)	4.44 (0.66)	0.38	0.29	2.06 (0.18)	-1.22 (0.12)	0.06 (0.09)	1.08 (0.11)	2.32 (0.21)	1.22	0.77
I8	1.27 (0.16)	-0.48 (0.12)	1.27 (0.17)	3.24 (0.41)	/	0.46	0.33	2.45 (0.21)	-0.95 (0.10)	0.13 (0.09)	1.11 (0.10)	2.29 (0.20)	1.70	0.96
I11	1.65 (0.19)	0.01 (0.10)	1.44 (0.15)	2.58 (0.28)	3.82 (0.55)	0.81	0.49	2.44 (0.21)	-0.79 (0.09)	0.14 (0.09)	1.19 (0.11)	2.30 (0.20)	1.70	0.93
I24	1.46 (0.19)	0.64 (0.11)	1.92 (0.22)	3.09 (0.38)	4.17 (0.65)	0.65	0.36	2.67 (0.24)	-0.42 (0.08)	0.20 (0.09)	1.33 (0.11)	2.39 (0.22)	2.11	1.00
I25	1.03 (0.13)	-1.23 (0.19)	0.73 (0.15)	2.28 (0.29)	4.17 (0.60)	0.32	0.26	1.93 (0.18)	-1.04 (0.12)	-0.17 (0.09)	1.06 (0.11)	2.31 (0.21)	1.11	0.68
I4	1.85 (0.23)	0.51 (0.09)	1.92 (0.22)	3.91 (0.65)	/	0.93	0.49	2.90 (0.26)	-0.65 (0.08)	0.22 (0.09)	1.30 (0.10)	2.46 (0.23)	2.33	1.19
I13	1.93 (0.26)	1.00 (0.12)	2.10 (0.22)	3.45 (0.49)	/	1.07	0.50	3.06 (0.27)	-0.48 (0.08)	0.30 (0.09)	1.38 (0.11)	2.50 (0.24)	2.61	1.26
I16	2.29 (0.25)	0.20 (0.08)	1.50 (0.14)	2.39 (0.23)	/	1.52	0.67	2.89 (0.26)	-0.62 (0.08)	0.22 (0.09)	1.42 (0.11)	2.38 (0.21)	2.31	1.17
I17	2.02 (0.23)	0.09 (0.09)	1.44 (0.14)	2.41 (0.24)	3.32 (0.44)	1.22	0.67	2.51 (0.22)	-0.79 (0.09)	0.24 (0.09)	1.32 (0.11)	2.91 (0.34)	1.75	1.02
I20	2.24 (0.27)	0.63 (0.09)	1.94 (0.18)	2.98 (0.35)	/	1.41	0.65	2.99 (0.27)	-0.56 (0.08)	0.23 (0.08)	1.42 (0.11)	2.43 (0.22)	2.49	1.22
I3	2.34 (0.25)	0.02 (0.08)	1.30 (0.12)	2.33 (0.21)	3.12 (0.38)	1.61	0.84	3.94 (0.37)	-0.71 (0.08)	0.17 (0.08)	1.27 (0.09)	2.05 (0.16)	4.10	1.73
I5	2.89 (0.39)	1.00 (0.10)	1.78 (0.16)	/	/	2.33	0.59	3.65 (0.33)	-0.51 (0.08)	0.34 (0.08)	1.37 (0.10)	2.18 (0.18)	3.57	1.56
I12	2.44 (0.26)	0.08 (0.08)	1.17 (0.11)	2.11 (0.19)	3.03 (0.36)	1.71	0.89	3.64 (0.33)	-0.68 (0.08)	0.27 (0.08)	1.31 (0.10)	2.10 (0.17)	3.56	1.57
I23	2.55 (0.32)	0.90 (0.10)	1.65 (0.15)	2.71 (0.28)	/	1.90	0.72	4.71 (0.47)	-0.08 (0.07)	0.42 (0.08)	1.59 (0.11)	2.16 (0.18)	6.13	1.98
I26	2.98 (0.40)	1.01 (0.10)	1.86 (0.16)	2.83 (0.32)	/	2.47	0.90	4.76 (0.49)	0.01 (0.07)	0.41 (0.08)	1.58 (0.11)	2.16 (0.18)	6.52	1.94
I2	0.94 (0.13)	-0.74 (0.17)	1.05 (0.18)	3.58 (0.51)	5.88 (1.06)	0.26	0.21	2.72 (0.23)	-0.99 (0.10)	0.05 (0.08)	1.02 (0.10)	2.10 (0.17)	2.05	1.10
I6	0.97 (0.13)	-1.26 (0.21)	1.02 (0.17)	3.81 (0.54)	5.71 (1.01)	0.27	0.23	2.94 (0.25)	-1.02 (0.10)	0.06 (0.08)	0.95 (0.09)	2.13 (0.17)	2.38	1.23
I14	0.89 (0.12)	-1.44 (0.23)	0.51 (0.15)	2.64 (0.37)	4.70 (0.73)	0.24	0.21	2.28 (0.20)	-1.08 (0.11)	0.01 (0.09)	0.93 (0.10)	2.31 (0.20)	1.51	0.88
I22	1.00 (0.14)	-0.78 (0.16)	1.16 (0.19)	4.39 (0.67)	6.22 (1.26)	0.29	0.22	2.77 (0.24)	-0.76 (0.09)	0.05 (0.08)	1.12 (0.10)	2.24 (0.19)	2.16	1.10
I7	1.77 (0.18)	-0.56 (0.11)	0.80 (0.11)	2.34 (0.23)	3.21 (0.38)	0.94	0.59	3.04 (0.26)	-0.98 (0.09)	0.04 (0.08)	0.92 (0.09)	2.33 (0.20)	2.54	1.29
I9	2.55 (0.26)	-0.25 (0.08)	1.14 (0.11)	2.10 (0.18)	2.99 (0.35)	1.86	0.98	3.08 (0.27)	-0.89 (0.09)	0.11 (0.08)	1.18 (0.10)	2.52 (0.24)	2.54	1.33
I10	2.25 (0.24)	-0.10 (0.08)	1.19 (0.12)	2.25 (0.21)	3.46 (0.52)	1.43	0.82	3.31 (0.29)	-0.76 (0.08)	0.21 (0.08)	1.14 (0.09)	2.34 (0.21)	2.94	1.43
I15	2.14 (0.22)	-0.46 (0.09)	1.05 (0.11)	2.35 (0.22)	3.56 (0.54)	1.27	0.79	3.75 (0.33)	-0.74 (0.08)	0.16 (0.08)	1.10 (0.09)	2.17 (0.17)	3.73	1.66
I18	2.11 (0.22)	-0.20 (0.09)	1.21 (0.12)	2.17 (0.20)	3.06 (0.36)	1.32	0.74	3.57 (0.32)	-0.74 (0.08)	0.18 (0.08)	1.14 (0.09)	2.49 (0.25)	3.39	1.59
I19	1.91 (0.20)	-0.70 (0.11)	1.06(0.12)	2.51 (0.25)	/	0.99	0.60	3.66 (0.33)	-0.81 (0.08)	0.14 (0.08)	1.14 (0.09)	2.34 (0.21)	3.52	1.64
I21	2.07 (0.20)	-0.88 (0.11)	0.69 (0.10)	1.96 (0.18)	2.78 (0.30)	1.27	0.76	3.57 (0.31)	-0.86 (0.09)	0.09 (0.08)	1.07 (0.09)	2.05 (0.16)	3.38	1.56

"/"indicates that only four options are selected and the other option is not selected

*TIF* Test information function

^a^Mean test information function of seven categories, that is, categories − 3, − 2, − 1, 0, 1, 2, 3

Of the items, 89% and 100% (in the third trimester and at six-weeks postpartum, respectively) showed very high discrimination (≥ 1). The *β*_*ik*_ values for all items were between -1.44 (item 14) and 6.22 (item 22) in the third trimester and between -1.22 (item 1) and 2.91 (item 17) at six-weeks postpartum, indicating a broad range of information at both time points. The results also showed no disordinal or reversal of *β*_*ik*_. Moreover, TIF maximum values ranged from 0.178 (item 2) to 2.81 (item 16) in the third trimester and from 1.110 (item 25) to 6.515 (item 26) at six-weeks postpartum. Further, most (92.30%) item characteristic curves were well shaped; the peaks of the five curves did not overlap; moreover, curves 2, 3, and 4 were normally distributed (Appendices A and B).

## Discussion

To the best of our knowledge, this is the first study to validate the LEIDS-RR-CV among a sample of perinatal women. We conducted a comprehensive psychometric evaluation based on CTT and IRT of the LEIDS-RR-CV that confirmed the predictive effect of CR on depression and that the scale has satisfactory structural, criterion-related, known-group, and predictive validity, robust internal consistency, and test–retest reliability. Therefore, the 26-item LEIDS-RR-CV is a valid and reliable scale to quantify CR among perinatal women in China and can be used to identify women at high risk of depression during perinatal periods.

In our study, the LEIDS-RR-CV showed a Cronbach’s alpha, ICC, and marginal reliability with values > 0.7, indicating satisfactory internal consistency and temporal stability. Further, through CFA, we confirmed that a five-factor structure provided a good fit to the data and that all items were significantly loaded to the expected potential factors, similar to the results of the original version of the scale [21] and those of the Chinese version, which was validated among undergraduate students and women in remission from depression [23, 29]. However, our results differ from those of the Spanish version of the LEIDS-RR, which presented a four-factor model [22], and the Persian version, which involved a sample of patients with depression from the general population and showed a six-factor model [15].

Therefore, our findings extend the scientific evidence of applying the LEIDS-RR in different samples and cultural contexts. Specifically, we showed that the LEIDS-RR-CV could be used to evaluate CR and its subscales (i.e., hopelessness/suicidality, acceptance/coping, aggression, control/perfectionism, and avoidant coping) among perinatal Chinese women.

Consistent with previous studies [30–32], our findings further confirm the relationship between CR and depression among perinatal women; that is, the total score for CR was higher among women with depression than among those without depression. This might be explained by Teasdale’s differential activation hypothesis [12]. Compared with women without depression, women with a history of depression have a higher degree of negative cognitive change after experiencing minor sadness and emotional change, that is, a higher level of CR and a higher likelihood of depression relapse [33]. However, the results of the IRT analysis showed that the LEIDS-RR-CV has high discrimination and performed well over diverse individuals with low and high CR [34]. The findings suggest that the LEIDS-RR-CV has high discrimination and is expected to perform well when applied to perinatal women with various levels of CR.

We observed a significant correlation between our data for the LEIDS-RR-CV and the scores for the EPDS, indicating good criterion-related validity. We used the LEIDS-RR-CV to predict future depression across the perinatal period, which showed acceptable test performance (i.e., AUC values > 0.7) [23]. Therefore, the 26-item LEIDS-RR-CV can identify women at high risk of depression and predict future depression during the perinatal period. Therefore, it is clinically useful for stakeholders interested in accurately assessing CR in this population.

To the best of our knowledge, our study is the first to attempt to establish optimal cut-off scores on the LEIDS-RR-CV among perinatal women; the optimal assessment scores were 58 and 60 points for the third trimester and at six-weeks postpartum, respectively. Therefore, women assessed in the third trimester, showing a total score of CR ≥ 58, were at a higher risk of depression than those with results below this value; at six-weeks postpartum, this cut-off value was ≥ 60. Specifically, our results show that the cut-off score for this scale varied according to the particular context of individuals [35]. We suggest that future scholars explore how the LEIDS-RR-CV performs when used in different clinical settings and subgroups. Our findings on the optimal cut-off score of CR among perinatal women may improve the ability of healthcare professionals to quickly identify patients with depressive thoughts and those at risk of clinical PND. Knowledge of this index may also prove helpful in developing referral, intervention, and treatment strategies to reduce the incidence of PND.

### Limitations

First, this research uses convenience sampling, wherein all women in our sample were recruited from Fujian province, southern China. Further, data were collected only through online self-reported questionnaires, which may have introduced sample bias and affected the generalizability of our results. Second, there is a need for additional evidence of the scale’s validity, particularly regarding its measurement invariance. Therefore, differential item function analysis may be an important step to confirm that the items in the scale function are similar across different groups of women. Finally, although we established a cut-off value for CR to identify groups of women at high risk of depression at both the third trimester and six weeks postpartum, we have not defined the cut-off values for mild, moderate, and severe CR scores, which future research should consider.

### Implications

This study attempts to develop a valid tool for indirectly predicting depression to further aid healthcare providers or public health service providers manage, specifically by prevention, PND in China, where there seems to be no such tool. We recommend testing and applying the LEIDS-RR among a larger representative group of perinatal women in different countries and comparing CR between western and Chinese groups. The LEIDS-RR will facilitate the design of psychological interventions aimed at curbing CT among perinatal women and is crucial to the effective development and evaluation of interventions to decrease CR, even PND, in the cross-cultural context.

## Conclusions

This study extends the psychometric analysis and scientific evidence of the applicability of the LEIDS-RR-CV among perinatal Chinese women. The scale showed adequate reliability and validity and can be used to evaluate CR to predict PND among perinatal women and to identify women at high risk of depression.

## Supplementary Information

**Additional file 1.** Illustration of item characteristic curves, and test information function for all items in the third trimester. Illustration of item characteristic curves, and test information function for all items at 6-week post-partum.

## Data Availability

The datasets used and/or analysed during the current study are available from the corresponding author on reasonable request.
