# Supplementary material for: Supporting routine cognitive reactivity assessment during the perinatal period: psychometric testing of the Chinese version of the Leiden Index of Depression Sensitivity
Source: BMC Pregnancy Childbirth. 2022 Dec 6;22:911. doi: 10.1186/s12884-022-05233-6 (PMC9727893; doi:10.1186/s12884-022-05233-6)
Supplement: Supplementary file 1 — Additional file 1. Illustration of item characteristic curves, and test information function for all items in the third trimester. Illustration of item characteristic curves, and test information function for all items at 6-week post-partum. [file 12884_2022_5233_MOESM1_ESM.docx]

**
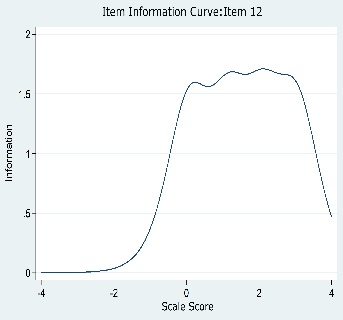
**
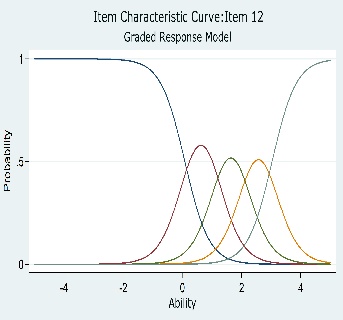
**
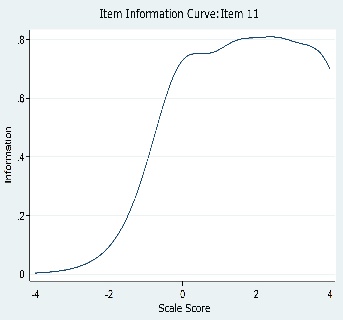
**
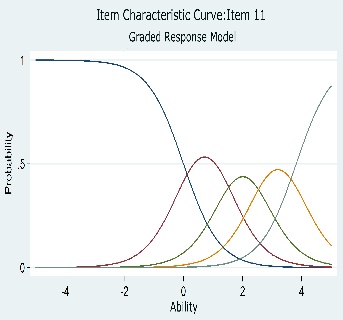
**
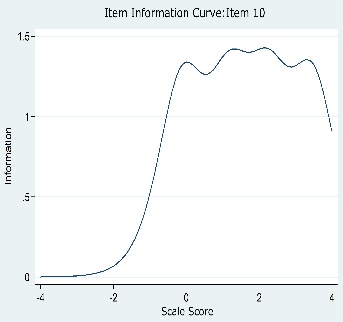

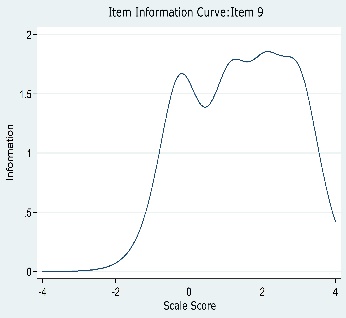
**
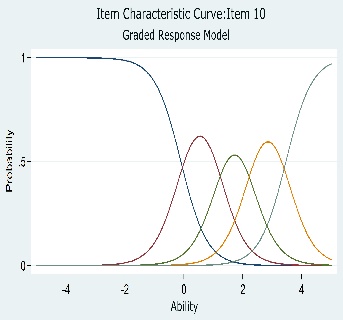

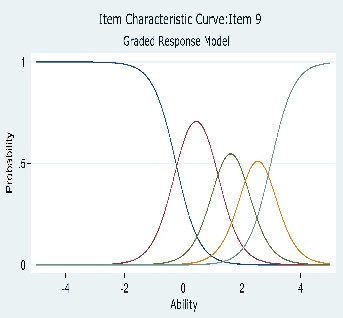

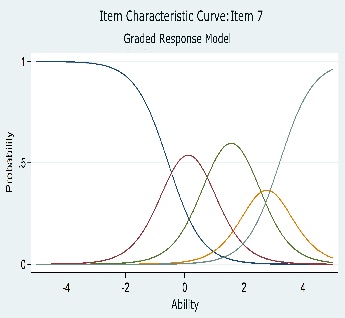
**
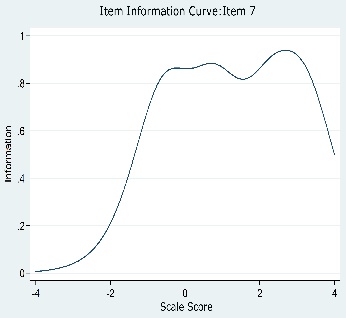
**
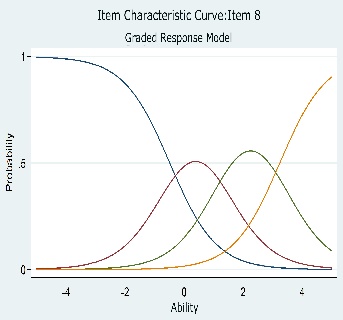

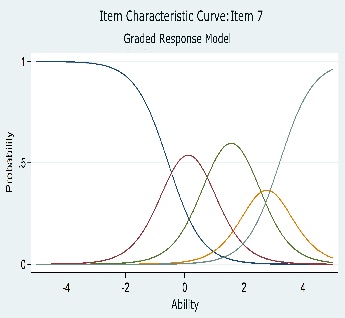
**
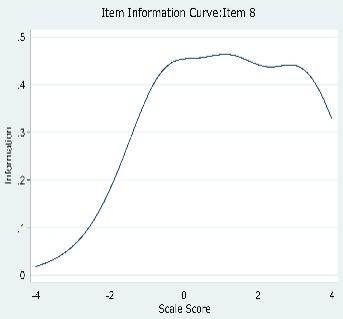
**
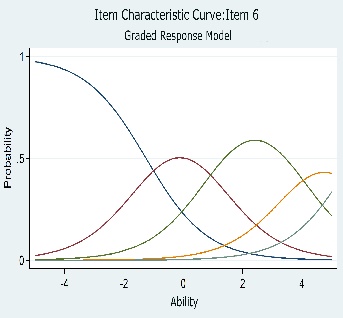

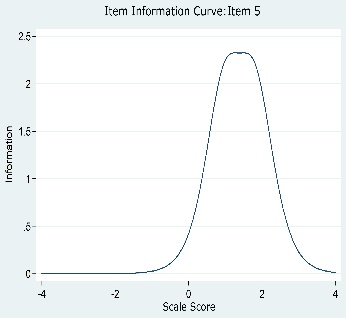

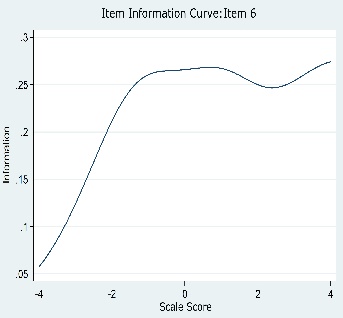

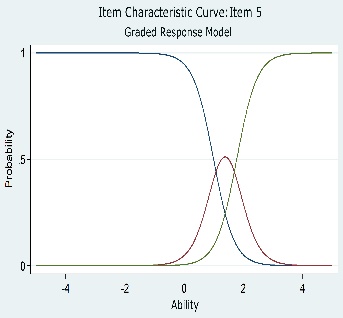

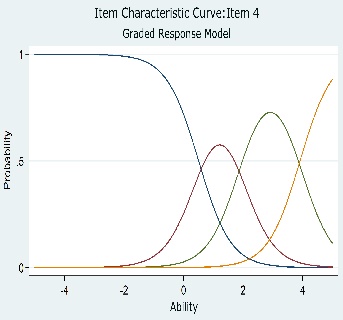
**
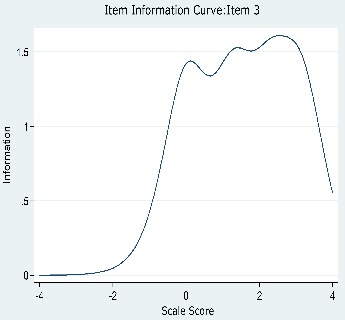

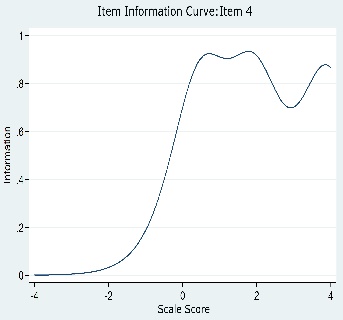
**
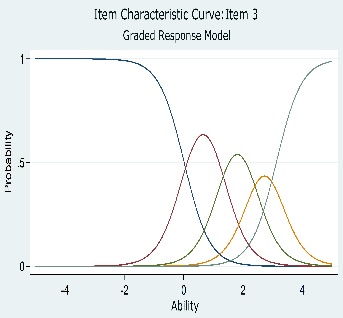
**
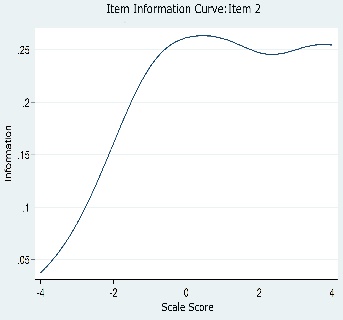

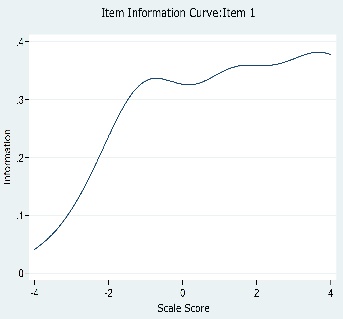
**
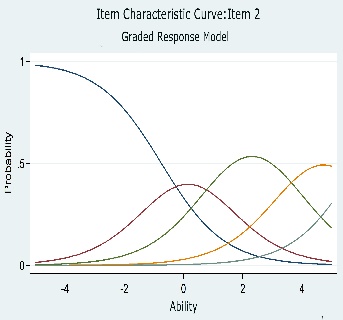

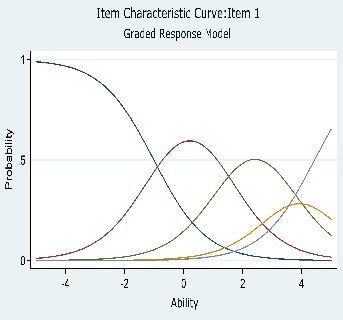


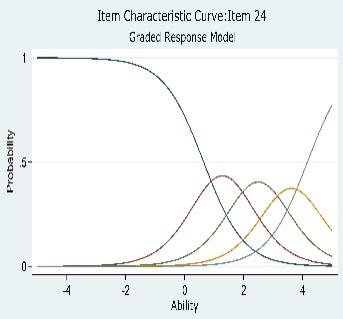

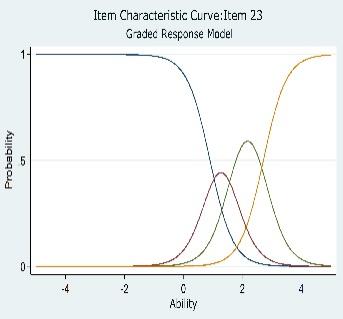

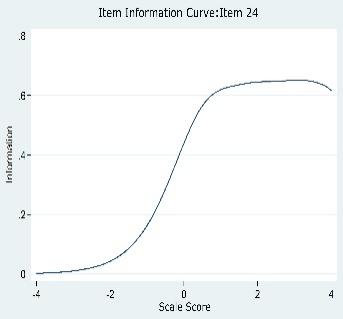

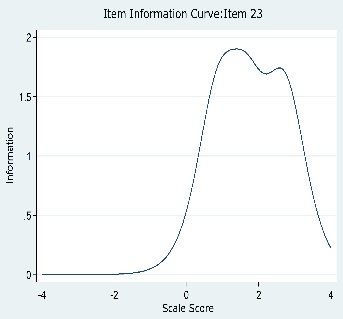

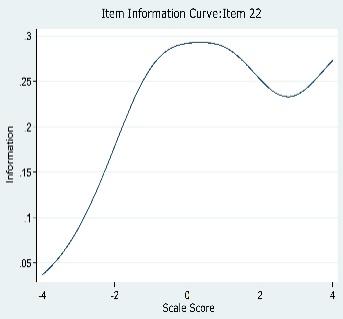

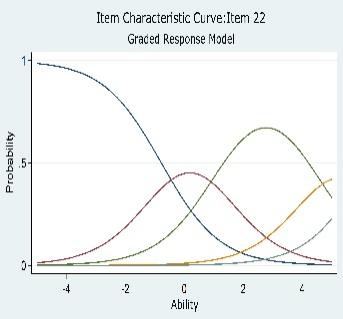

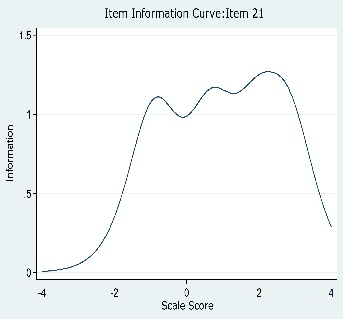

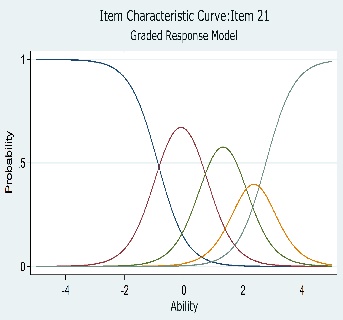
**
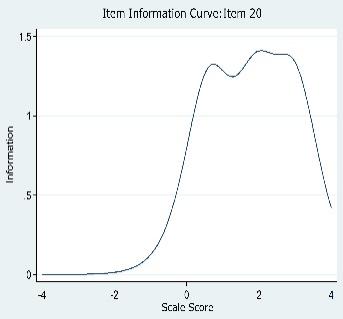
**
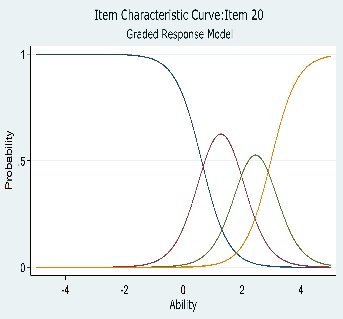

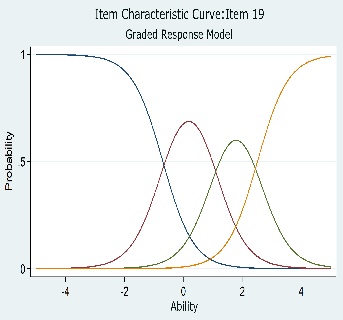
**
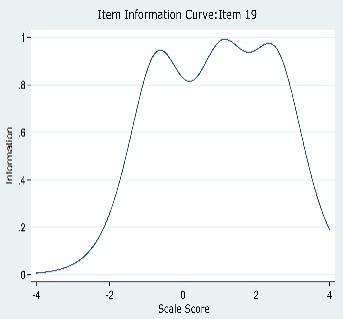

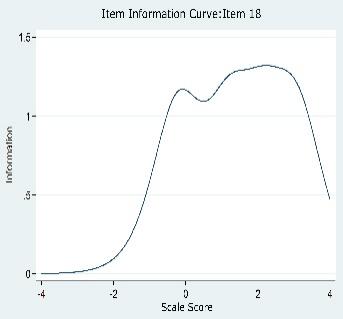
**
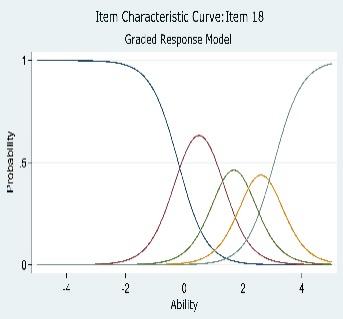

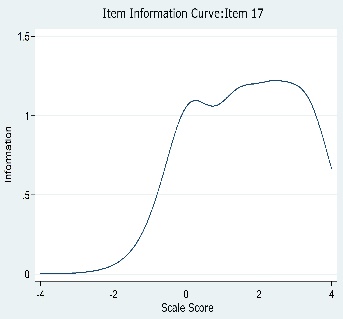

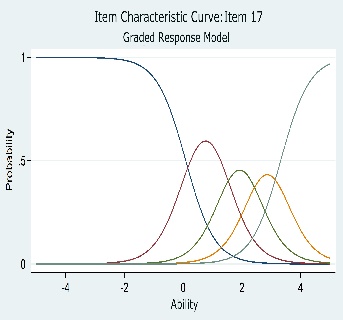
**
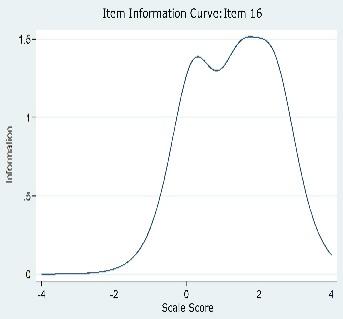
**
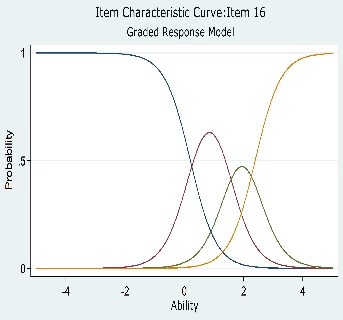

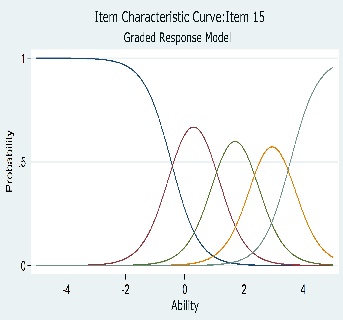
**
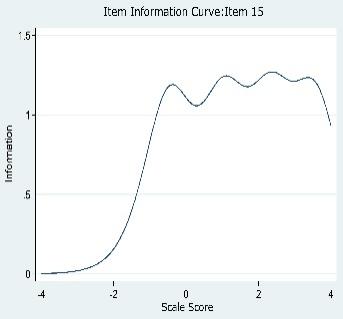

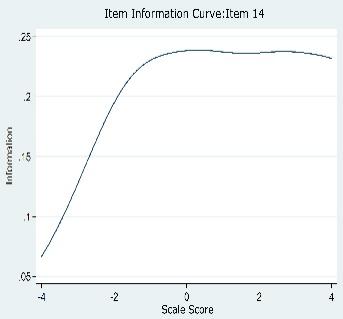
**
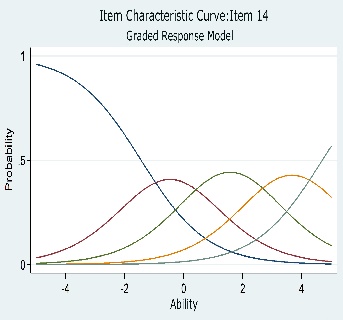
**
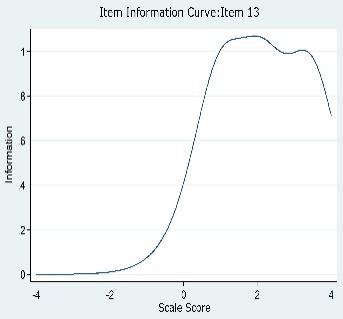
**
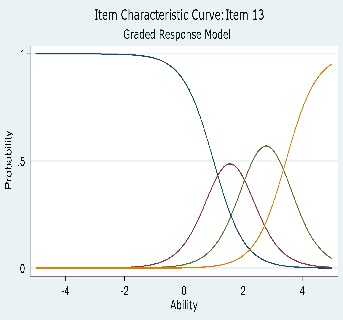


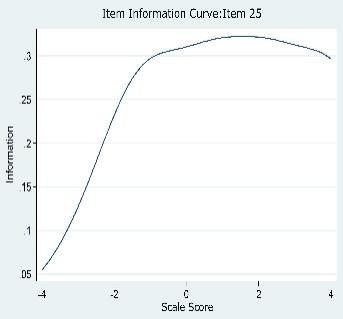

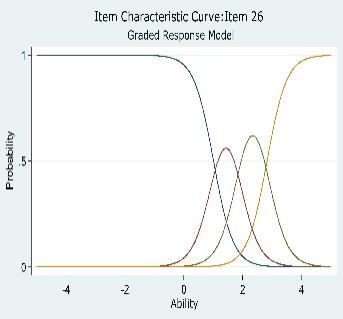

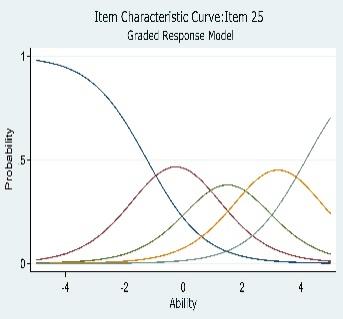

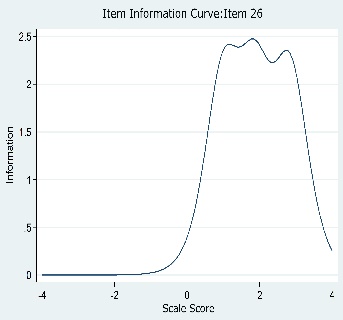


**Appendix A.** Illustration of item characteristic curves, and test information function for all items in the third trimester

Note. Curve 1,2, 3,4 and 5 represents the probability of a patient choosing each of the response options, 1 (not at all), 2 (a bit), 3 (moderately), 4 (strongly) and 5 (very strongly), respectively.


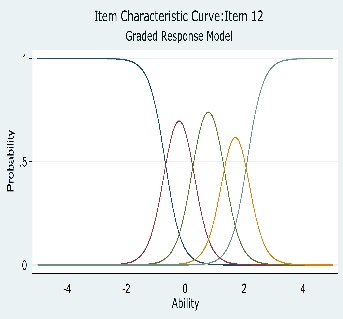
**
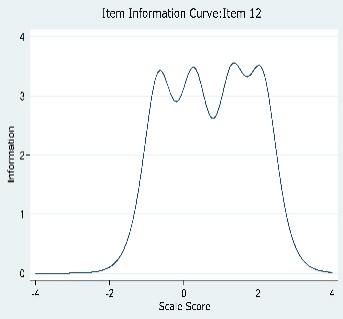

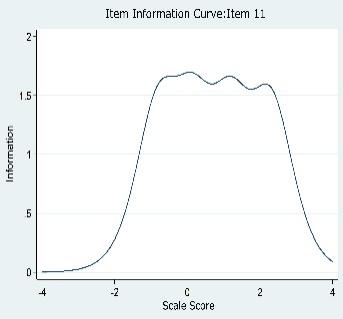
**
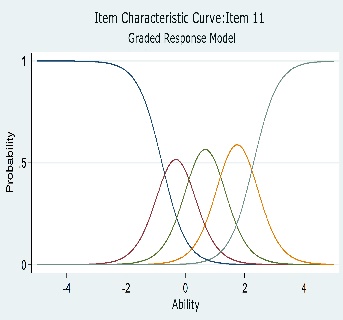

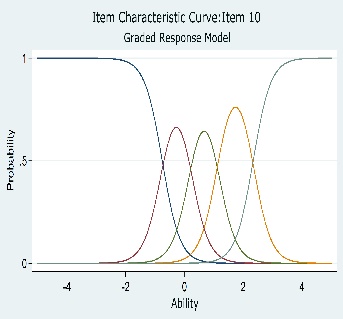

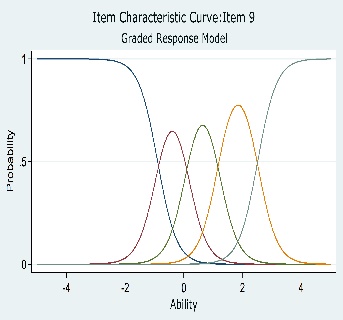
**
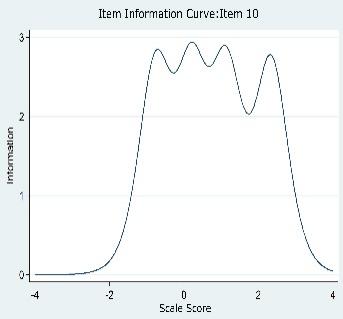

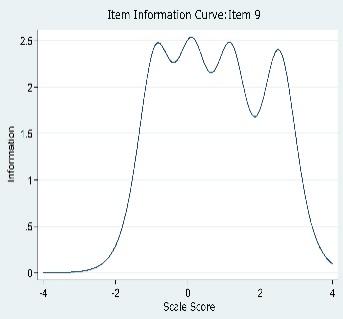

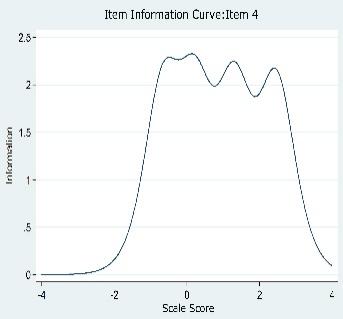

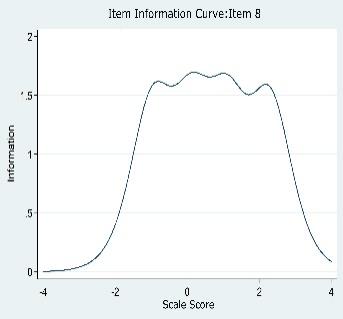

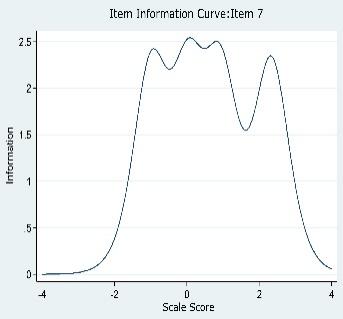
**
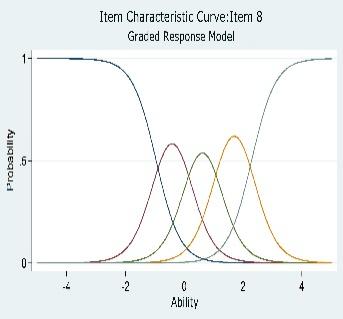

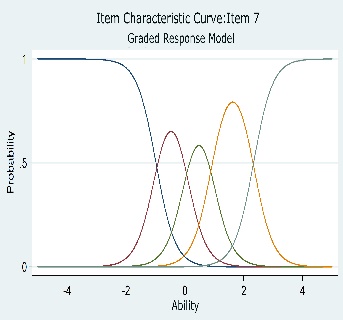
**
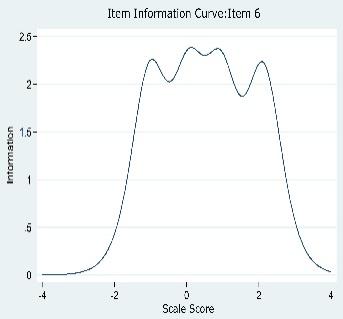

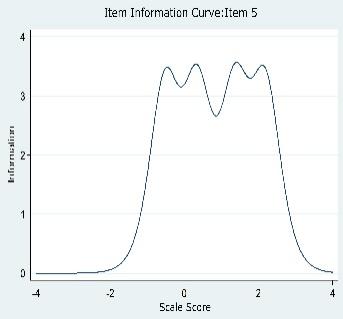
**
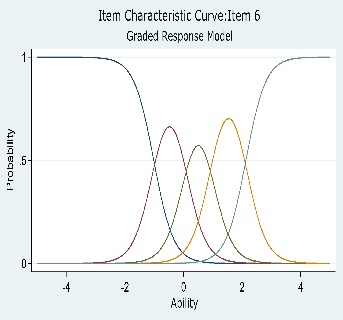

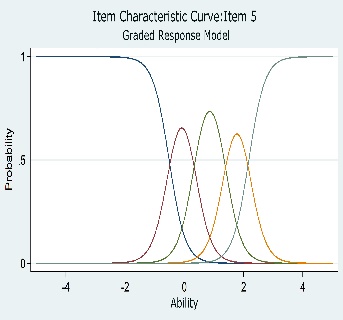

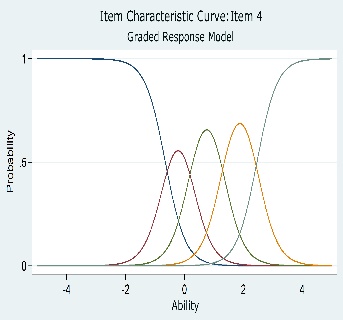
**
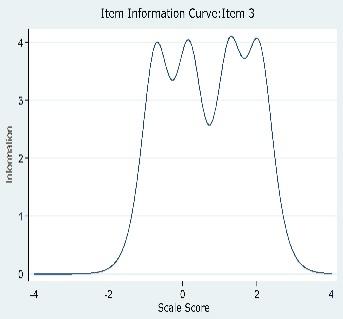
**
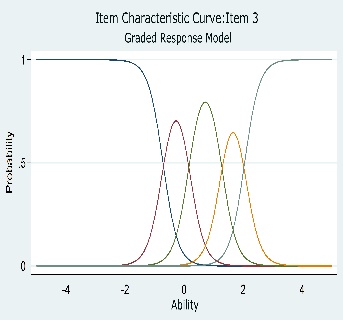
**
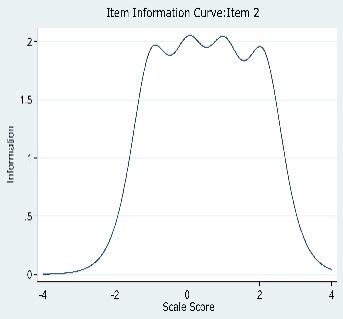
**
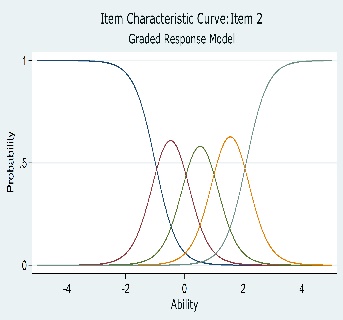
**
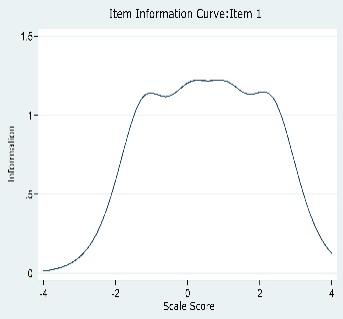
**
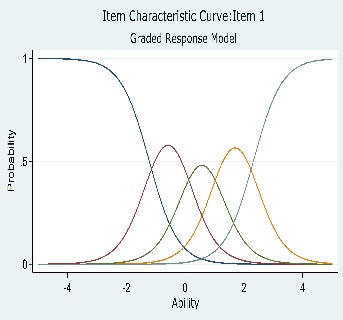


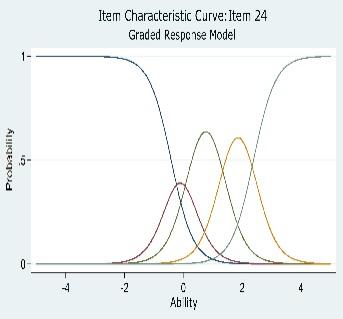

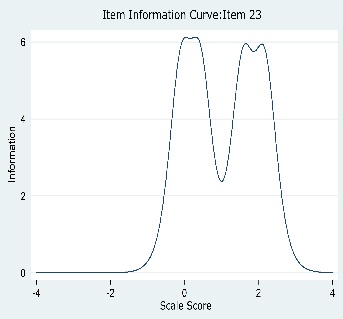

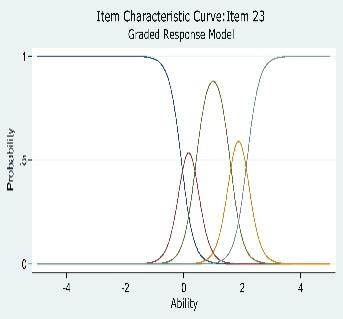

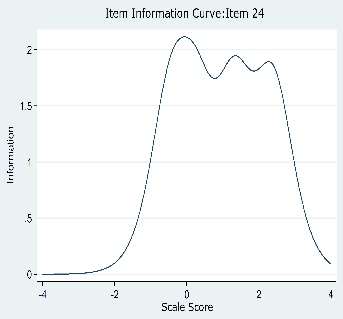
**
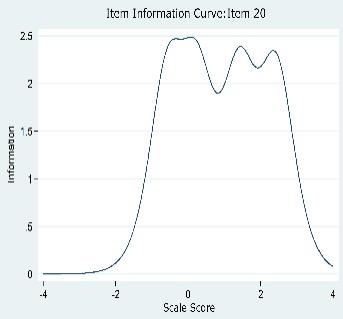
**
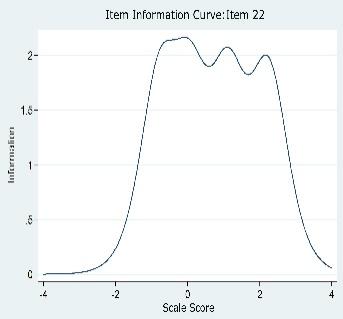

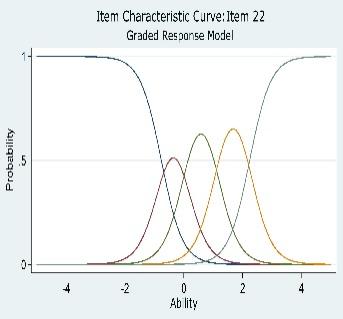

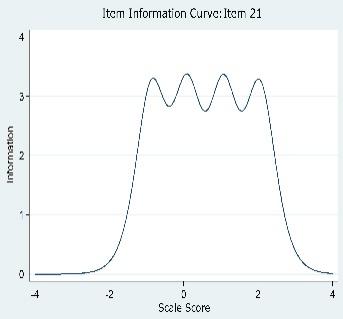

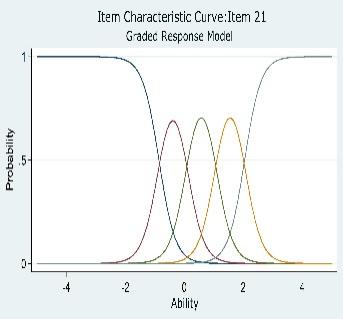

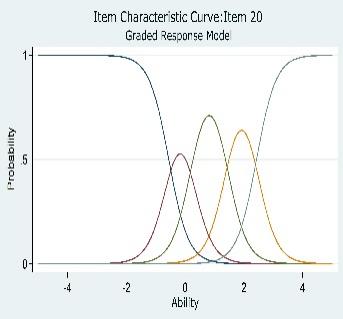
**
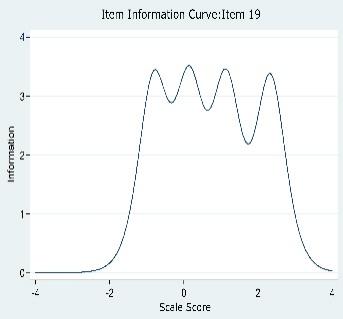
**
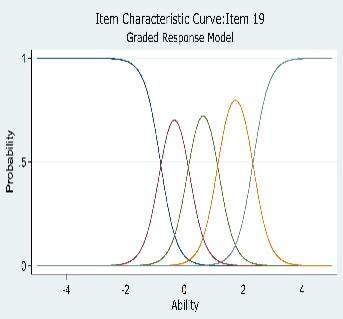
**
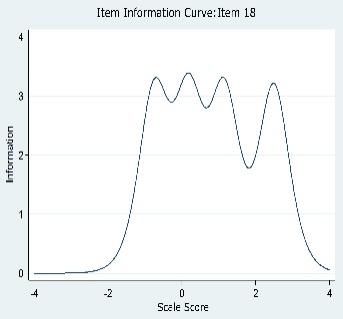

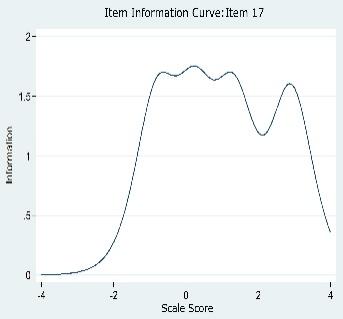
**
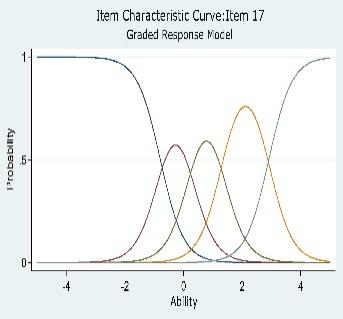

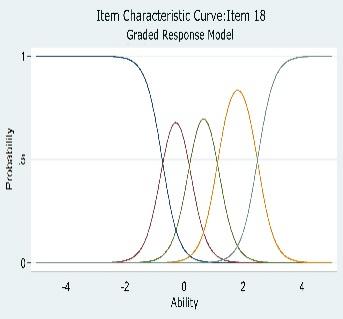

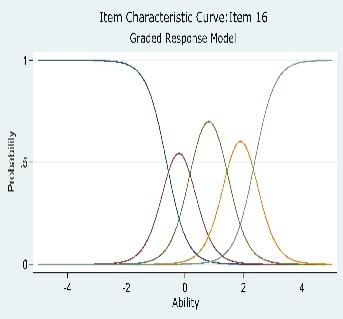
**
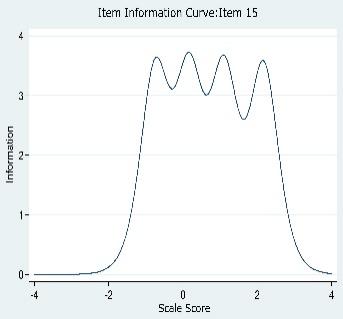
**
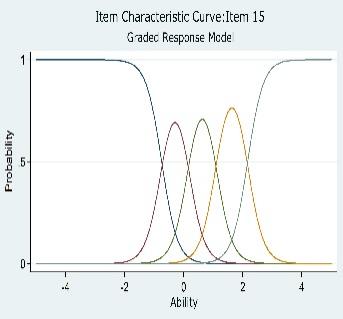
**
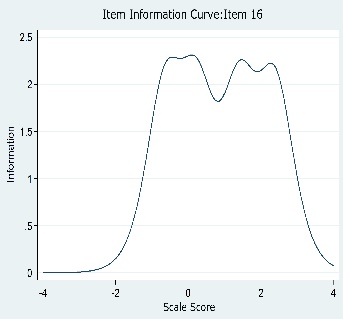

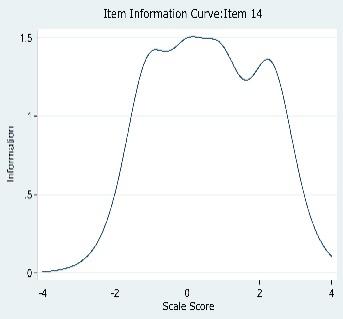

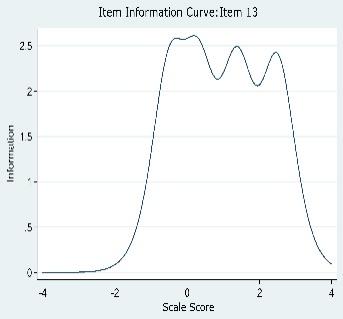
**
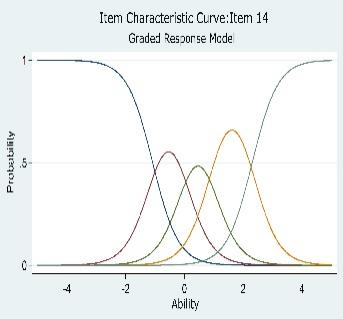


**AppendixB.** Illustration of item characteristic curves, and test information function for all items at 6-week post-partum.

Note. Curve 1,2, 3,4 and 5 represents the probability of a patient choosing each of the response options, 1 (not at all), 2 (a bit), 3 (moderately), 4 (strongly) and 5 (very strongly), respectively.
